# Human Cell Chips: Adapting DNA Microarray Spotting Technology to Cell-Based Imaging Assays

**DOI:** 10.1371/journal.pone.0007088

**Published:** 2009-10-28

**Authors:** Traver Hart, Alice Zhao, Ankit Garg, Swetha Bolusani, Edward M. Marcotte

**Affiliations:** 1 Center for Systems and Synthetic Biology, University of Texas at Austin, Austin, Texas, United States of America; 2 Department of Chemistry and Biochemistry, University of Texas at Austin, Austin, Texas, United States of America; 3 Institute for Cellular and Molecular Biology, University of Texas at Austin, Austin, Texas, United States of America; Brunel University, United Kingdom

## Abstract

Here we describe human spotted cell chips, a technology for determining cellular state across arrays of cells subjected to chemical or genetic perturbation. Cells are grown and treated under standard tissue culture conditions before being fixed and printed onto replicate glass slides, effectively decoupling the experimental conditions from the assay technique. Each slide is then probed using immunofluorescence or other optical reporter and assayed by automated microscopy. We show potential applications of the cell chip by assaying HeLa and A549 samples for changes in target protein abundance (of the dsRNA-activated protein kinase PKR), subcellular localization (nuclear translocation of NFκB) and activation state (phosphorylation of STAT1 and of the p38 and JNK stress kinases) in response to treatment by several chemical effectors (anisomycin, TNFα, and interferon), and we demonstrate scalability by printing a chip with ∼4,700 discrete samples of HeLa cells. Coupling this technology to high-throughput methods for culturing and treating cell lines could enable researchers to examine the impact of exogenous effectors on the same population of experimentally treated cells across multiple reporter targets potentially representing a variety of molecular systems, thus producing a highly multiplexed dataset with minimized experimental variance and at reduced reagent cost compared to alternative techniques. The ability to prepare and store chips also allows researchers to follow up on observations gleaned from initial screens with maximal repeatability.

## Introduction

Despite enormous progress in the postgenomic era, large-scale characterization of mammalian genes remains a daunting challenge. Classical perturbation experiments have been enabled by the creation of RNAi and chemical libraries, but there exist few platforms able to conduct cell-based experiments on the scale of mammalian genomes, especially when multiple reporters are required [1], [2]. Plate-based assays can be used for high-content screening of cell populations [3] or to capture detailed cell morphology and state information [4] – in fact a number of dedicated commercial platforms are on the market [5] – but these latter applications come at a high reagent cost relative to miniaturized assays. High-throughput flow cytometry probing immunolabeled phosphoproteins [6], [7] allows multiparameter sampling of protein activation state across a large cell population, but requires serial analysis of samples, hence performing sequential assays of every experimental condition or timepoint – a key limitation when performing genome-scale screens. Transfected cell microarrays [8]–[11], where cells are grown over a glass slide printed with transfection constructs, allows screening of expression [11] or RNA interference libraries [9] for functional genomics or drug screening [8], [10], and the technology allows multiple conditions to be tested on replicate slides. Transfected cell arrays have been applied to identifying genes involved in chromosome maintenance [12], measuring response of neural precursor cells to a variety of extracellular matrix components [13], finding ubiquitin targets [14], and even detecting protein-protein interactions [15]. For transfection-based screens, however, the technique is limited to cell lines compatible with the transfection technique used, although Sabatini and colleagues created arrays based on lentiviral infection constructs to circumvent this problem [16]. Tissue microarray (TMA) technology has enabled the multiplexed immunohistochemical analysis of tissue samples on a single array but thus far has been limited to tens or hundreds of samples per array [17]. Complementary technologies include cell lysate microarrays, in which the protein repertoire of a number of cell populations are spotted in parallel on a slide and assayed for reporters of cellular state [18], [19], although all data are population averages rather than per-cell readouts. Thus, there is a clear role for a platform that enables analysis of multiple cell types and/or treatment conditions in a manner that scales to thousands of samples, while minimizing reagent cost and experimental variance.

Here we describe the application of spotted cell microarrays to the study of human cell lines. Spotted cell microarrays, hereafter referred to as “cell chips,” are constructed by growing and treating cells under normal tissue culture conditions, formaldehyde fixing, and printing microsamples of each culture onto replicate glass slides. Each slide is then assayed by immunofluorescence against a specific target and imaged by high-throughput microscopy. Entire collections of cells comprising hundreds to thousands of discrete samples can be assayed onto replicate slides. Each slide is probed with a single reporter in a single assay, reducing experimental variance compared to multiwell plate assays where each well is effectively an independent experiment. Reagent cost is similarly reduced, with less than 100 uL of diluted antibody sufficient to probe a slide, 10–100 fold less than that required for a single 96-well plate assay. Importantly, cells from a wide variety of cell types (including both suspension and adherent cells), growth conditions, and treatments, can be arrayed on a single slide. Replicate slides allow researchers to conduct multiple assays against samples drawn from the same collection of treated cells, and to probe multiple pathways elements from the same sample of cells. Finally, slides can be stored after printing to facilitate generating replicates and following up on observations gleaned from initial screens with subsequent assays against samples drawn from the same original population of cells.

## Materials and Methods

### Cell culture

A549, HEK293, HeLa, DG-75, and Jurkat cell lines were acquired from ATCC. HeLa and HEK293 cells were maintained in DMEM medium, A549 in F12-K, and DG-75 and Jurkat in RPMI, each supplemented with 10% FBS (Gibco/Invitrogen). Cells were treated in culture flasks with staurosporine (Sigma), TNF-alpha (Sigma), or anisomycin (Sigma) as described.

### Printing cell arrays

To prepare cells for printing, adherent cell lines were washed with PBS, trypsinized (0.25% Trypsin in EDTA, Invitrogen) until detached (typically 2–5 min), and resuspended in PBS. Adherent and suspension cells were fixed in ¼ vol 10% fresh paraformaldehyde (Sigma) (2% final concentration) for 10 min, then washed in fresh PBS and transferred to a 1.5 ml Eppendorf tube for ease of handling.

After fixation, cells were treated with biotinylated wheat germ agglutinin (WGA-biotin; Biomeda) at 4 µg/ml final concentration for 15′, then washed 3x in PBS. Finally, cells were pelleted, media was removed, and cells were resuspended in 100% methanol at −20°C. Cells were incubated at least 10′, and can be stored at this stage for at least several weeks at −20°C.

Immediately prior to printing, cells were pelleted, MeOH was removed, and cells were resuspended in a minimum volume of PBS. A 20–50 µl of high-density cell suspension was transferred to a 384-well plate. Cells were allowed to settle to the bottom of the well, forming a loose pellet, before printing was initiated. Cells were printed on streptavidin-coated slides (TeleChem) with a custom-built DNA microarray printing robot [20] using blunt-tipped, slotted steel custom microarray pins (Majer Precision, part no. 11077-3 with a custom .003″ slot width). Printed slides were assayed immediately or stored at 4° or −20°C for several weeks.

### Assaying printed slides

Immunofluorescence labeling of target proteins was performed using antibodies against cleaved caspase 3, phospho-STAT1, phospho-JNK, phospho-p38, cleaved PARP, phospho-p65 (RelA) (Cell Signaling Technologies), p65/RelA, and histone H3 (Santa Cruz Biotechnology). First, a 16-well rubber gasket (Grace Biosystems) was trimmed to provide a single large reservoir on the slide around the array. The cells were blocked in 5% goat serum (Sigma) in PBS (30′, RT), washed once with PBS, and incubated 2 h (RT) or overnight (4°) with primary antibody diluted to manufacturer's specification in PBS +0.2% Triton X-100. Slides were washed in PBS 3x for 5′ ea in coplin staining jars, then incubated with goat anti-rabbit (or anti-mouse, as necessary) IgG conjugated with Alexa Fluor 594 (Invitrogen; diluted 1∶1000) for 60′ at RT in the dark. Slides were washed again 3x in PBS, with the final wash containing a 1∶10,000 dilution of Hoechst 33342 (Invitrogen). Slides were air-dried, mounted with ProLong Gold mounting medium and coverslipped before imaging.

Imaging was performed on a Nikon TE2000 microscope with motorized XY stage and Z objective and a Photometrics Cascade II 16-bit CCD camera. Using the NIS Elements controller software, we generated a script that would automatically visit each spot, autofocus (in DIC), and capture fluorescent images for Hoechst 33342 (nuclear) and immunostained labels. Imaging was performed using a 40x (0.95 NA) dry objective, and exposure times were selected to minimize the occurrence of saturated pixels under normal assay conditions.

Quantitation was carried out using Matlab and the Image Processing Toolkit. For each spot, depending on the region of interest (nuclear or whole-cell) for the given probe, either the image of the nuclear label or the one of the immunolabel was background corrected and converted to a binary pixel mask. The mean signal intensity in the immunofluorescence channel of all pixels within the mask is calculated and recorded for each spot, along with the number of pixels in the mask.

We discovered a pronounced linear bias in average pixel intensity that correlated with the number of pixels in the mask – which itself reflects the number of cells deposited in the spot. A correction for the size bias was applied by normalizing signal to that found with wildtype (untreated) cells. A linear regression is applied to signal derived from wildtype cells, and the signal of a given spot is then measured as a distance from the regression line at the same mask size of that spot. This method is effective as long as the range of wildtype mask sizes meets or exceeds the range of mask sizes for treated cells; this was true for all cases discussed here.

The bias-corrected mean signal intensity was determined for each spot of treated cells. These were compared to spots of untreated cells of the same type on that slide, yielding a distinct set of data for each cell type, even where multiple cell types are arrayed on a slide (bias-corrected mean signal intensity data are shown in Table S1). Treated and untreated spots were compared by unpaired one-tailed T-test for samples with different variances. By definition, the bias-corrected mean sample intensity of control spots is zero.

## Results

**Figure 1:**
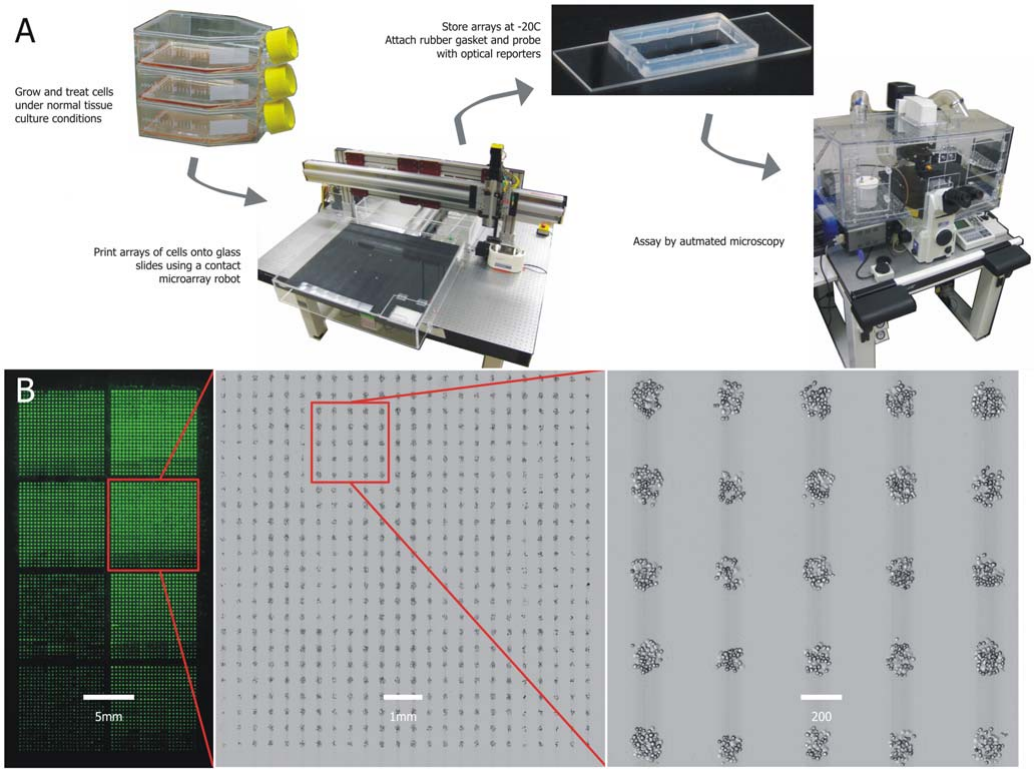
Overview of spotted cell chip process. (A) Cells are grown and treated under normal cell culture conditions. Our recommended protocol (less successful early protocols are discussed in the text and in Fig. 2) involves trypsinizing adherent cells and fixing with formaldehyde, decorating with WGA-biotin, and permeabilizing in −20°C methanol. Cells can be stored for several weeks in this state before resuspending in PBS and transferring to source plate for printing. Using a robotic microarray spotting device, cells are printed onto streptavidin-coated slides. To assay, each slide is probed by immunofluorescence against the target of choice and imaged by automated microscopy. (B) A high-density cell chip. An 8-pin print of 4,608 replicate spots, each containing a microsample of HeLa cells, is shown by imaging with a microarray scanner (left; green signal is light scattering in the fluorescent channel off freshly printed spots) and by stitching together multiple 10x microscope images (center and right).

Spotted cell microarrays were first developed and applied to functional genomic screens in *Saccharomyces cerevisiae* [21] and bacteria [22]. To print yeast cell chips, we used a contact microarray printing robot to draw a microsample from a suspension of fixed cells in a 96-well microplate (the “source plate”) and deposit it on a poly-L-lysine coated glass slide. To print human cell lines, we used custom microarray pins with blunt tips and wide slots, and after experimenting with other adhesion protocols, we determined that printing biotin-decorated cells on streptavidin-coated glass slides ensured cell adhesion and reproducibility. An overview of the process is shown in **Figure 1a**. To demonstrate achievable array densities, we printed eight replicate HeLa cultures repeatedly onto a slide. A total of 4,608 spots were successfully printed on a single slide (**Figure 1b**), using eight spotting pins and a spot pitch of 400 µm. Chips of much higher density, exceeding 8,000 spots per slide, could be achieved by decreasing spot pitch ∼10% and increasing to 12 spotting pins.

Contact microarray technology, typically used to print DNA oligonucleotides or cDNA sequences for use in RNA hybridization assays, is optimized around printing the smallest spots that can be consistently delivered. During the development of the yeast spotted cell microarray technique it was observed that better performance was achieved using microarray pins that had been “blunted” by repeated use in printing cDNA arrays. The blunted pins gave a larger spot size, a greater volume of medium deposited and, typically, a larger number of cells in the spot. However, the degree of blunting and therefore the quality of spots delivered varied widely among these well-used pins.

To adapt cell chips to human cells, we initially used the same microarray pins as in the yeast cell chip, and printed on poly-L-lysine (poly-K) coated slides. Early testing was conducted using the Jurkat T-cell leukemia cell line, as these cells are easy to grow in large quantities and a successful cell chip would provide a new platform for assaying suspension cells. We immediately observed that the larger human cells – which are typically spheroids 10–20 µm in diameter, many times larger than ovoid yeast cells that measure 3–8 µm on the long axis – did not print consistently onto poly-K coated slides, and that the inconsistency was in part attributable to how deformed the microarray pins were. To address this issue in a more systematic manner we acquired microarray pins with sharp or blunt tips in three sizes (Majer Precision MicroQuill 2000, part nos. 11077-1, 11077-2, and 11077-3). The 11077-1 pins were sharp and yielded spots <100 um in diameter, while the -3 pins had the largest blunt area and gave spots ∼200 um across. Quantity of cell deposition was further improved by using custom pins, based on the 11077-3 form factor, but with a slot width of 0.030″ (76 µm) vs. the standard 0.015″ (38 µm). The smaller slot is only 2–3 cell diameters in width and may have induced shear effects and clumping as cells were loaded and deposited by the pins; these effects appear to have been largely mitigated by using the wider slots. The custom 11077-3 pin with 0.03″ slot width consistently delivers a spot ∼200 µm in diameter and was used for all subsequent human cell chip prints.

Although we achieved regularity in spot sizes by selecting the appropriate microarray pins, the number of spots delivered was found to be highly dependent on the concentration of cells in the 384-well source plate. Depositing 50 cells in a spot ∼1 nl in volume implies a concentration of ∼50,000 cells/µl, or 10^6^ cells in 20 µl suspension in each well of the source plate. However, during the time required to print ∼100 samples onto each of 10–20 slides – roughly 30 minutes – the cell suspension settles into a loose pellet at the bottom of the well. In an effort to maintain the cells in suspension during printing, we increased the viscosity of print media using glycerol (15–50%) and sucrose (30–50%).

We tested the cell chip's ability to detect cellular state by inducing apoptosis in Jurkat cells. We grew the cells under normal tissue culture conditions. Separate cultures were treated with staurosporine, a potent inhibitor of protein kinase C and other essential cellular kinases, and fixed with formaldehyde after 1, 2, or 4 hours. Treated and untreated cells were collected in several wells of a 384-well plate at a concentration of >10^5^ cells/µl and printed on poly-L-lysine coated slides such that each sample was printed several times on each of several replicate slides.

**Figure 2:**
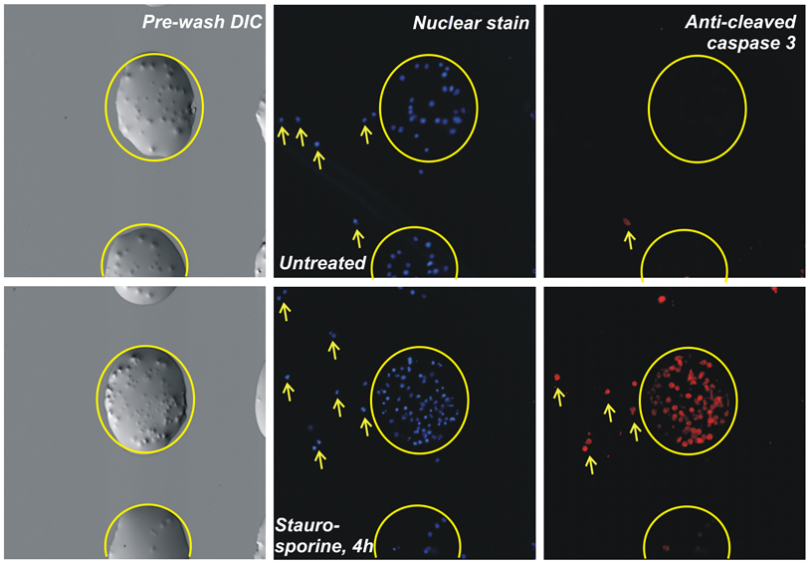
15X images of an early print of Jurkat cells onto poly-L-lysine slides without the use of WGA-biotin. Left panels: DIC images acquired immediately after printing. A 30% sucrose print buffer prevented complete liquid evaporation after printing. Other panels show nuclear stain (center) and immunofluorescence (right) against cleaved caspase 3, an indicator of apoptosis. Top row, untreated; bottom row, treated with staurosporine, 4 h. Yellow circles indicate one printed spot; arrows indicate cells outside the circle translocated during the immunofluorescence protocol. As a consequence, all subsequent prints were conducted with WGA-biotin-decorated cells printed on streptavidin-coated slides.

Immediately after printing, slides were imaged with transmitted light to analyze print quality; printed spots were discrete and typically contained 20–50 cells. Three slides were then probed for signs of apoptosis by immunofluorescence with antibodies against cleaved caspase 3, cleaved caspase 9, and cleaved PARP. Each slide was also labeled with a nuclear stain, and each spot was imaged using automated microscopy. Images of Jurkat cells immediately after printing, and of labeled cells after probing for cleaved caspase 3, are shown in Figure 2.

Although the immunofluorescence data supported the prototype cell chip's ability to detect cellular state, we observed that a significant number of cells – perhaps 10% of the cells in some spots — had shifted on the slide during the wash steps of the immunofluorescence protocol. This translocation is evident in Figure 2 when the pre-probe DIC images are compared to the fluorescent images (see arrows in figure). Given the relatively small numbers of cells in each spot, cross-contamination of even individual cells could dramatically reduce the dynamic range of the cell chip as an assay tool. To alleviate this problem, we tested an alternate adherence technique involving an adaptor molecule instead of relying on electrostatic interaction. After fixation, we decorated cells with a biotinylated lectin, wheat germ agglutinin (WGA-biotin), and printed the cells on streptavidin-coated slides. Under this protocol, increased print buffer viscosity is not required; cells were resuspended at 10^6^ cells in 20 µl PBS (without glycerol or sucrose) in each well of the 384-well source plate and allowed to settle into a loose pellet. The microarray robot was calibrated to dip the pins into the pellet during loading. We printed WGA-biotin-labeled Jurkat and DG-75 suspension cells as well as trypsinized HeLa and HEK293 adherent cells onto replicate chips. Using the wide-slot pins and a standard wash cycle between loads, we observed neither cell clumping in the pins nor cross-contamination of cells into adjacent spots. After printing on a streptavidin-coated slide and allowing the print to dry, we observed no cell translocation throughout many repeated washing steps. The WGA-biotin/streptavidin slide combination was used for all subsequent prints.

To demonstrate the multiplex capability of the cell chip, we printed chips with both A549 non-small-cell lung cancer cells and HeLa cervical cancer cells. Each cell line was divided into three cultures: one treated with anisomycin (1 µM, 30′), one with TNFα (10 ng/ml, 60′), and one untreated control. Anisomycin, a translation inhibitor, activates (by phosphorylation) both the p38 and c-Jun N-terminal kinase (JNK) stress kinases. Among the effects of TNFα exposure are JNK activation and NFκB translocation to the nucleus. NFκB is maximally concentrated in the nucleus at about an hour after TNFα exposure [23], while JNK activation peaks after about 15 minutes and degrades to background levels about half an hour later [24]. Multiple replicate chips were printed, each carrying all six conditions printed in multiple replicate spots.

**Figure 3:**
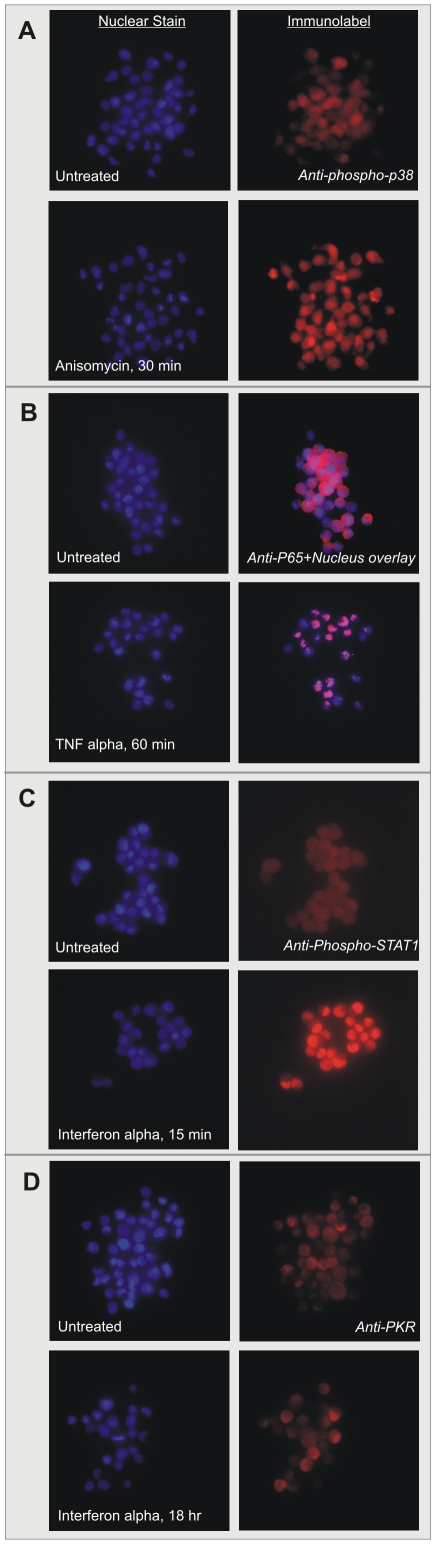
Cell chips can be printed with multiple cell types, treatments, and time points on the same arrays, enabling extensive multiplexing of experiments. (A) HeLa and A549 cells were grown in T-75 flasks collected after treatment with anisomycin, TNFα, or no treatment, and printed on replicate slides. A slide was probed for phosphorylated p38 kinase; images show Hoechst 33342 nuclear stain (left) and IF spots of treated and untreated HeLa cells. (B) A slide from the HeLa/A549 print was probed for the p65/RelA subunit of NFκB. Right panels show overlay of brightest pixels from IF images onto nuclear stain, demonstrating cytoplasmic localization in untreated cells and nuclear translocation in response to TNFα. (C) A549 cells were grown in T-75 flasks and collected without treatment and after treatment with interferon-alpha (1000 U/ml) for 15 minutes or 18 hours. A chip was probed for phospho-STAT1; sample images of one 15-minute timepoint and one untreated spot show strong difference in signal. (D) A slide from the same print was probed for PKR; a weak signal is detected in the 18 hour timepoint, which corresponds to a ∼3-fold increase in protein level as detected by Western blotting (data not shown).

Individual chips were probed for phospho-p38 kinase, phospho-JNK, and the p65/RelA subunit of NFκB. Each slide was counterstained with Hoechst 33342 nucleic acid stain and a high resolution image of each spot was captured in the corresponding fluorescent wavelengths. **Figure 3a** illustrates representative nuclear stain and immunofluorescence images from the chip probed for phospho-p38; the two spots show the increase in signal in HeLa cells treated with anisomycin compared to controls. The translocation of NFκB to the nucleus in response to TNFα in both cell lines was apparent in the images (**Figure 3b** shows p65 translocation in HeLa cells).

We analyzed the set of treated spots from each cell line for an increase in signal relative to that cell type's control spots on the same slide by comparing the set of mean bias-corrected signal intensities of each set of spots (two-sample one-tailed T-test; see Methods). Calculated p-values are shown in **Table 1**. JNK was phosphorylated in response to anisomycin treatment in both cell lines but TNFα-treated cells showed a weak response only in HeLa, consistent with the expected dynamics of TNF-induced JNK activation and deactivation. Anisomycin also activated p38 in HeLa cells, as expected, but surprisingly the response was much weaker in A549s; the p-value of 0.01 is not significant after multiple-hypothesis correction. P65 translocation to the nucleus is represented as an increase in nuclear signal in HeLa cells.

**Table 1:** Measuring cellular response to drug treatments (Anisomycin, 1 µM, 30′; TNFα, 10 ng/ml, 60′) on A549 and HeLa cells printed on the same chips.

Probe	A549								HeLa							
	Control		Anisomycin			TNFα			Control		Anisomycin			TNFα		
	<I>	SE	<I>	SE	Pval	<I>	SE	Pval	<I>	SE	<I>	SE	Pval	<I>	SE	Pval
p-P38	0 (n = 10)	397	491 (n = 5)	294	.01	252 (n = 4)	384	.13	0 (n = 10)	225	2256 (n = 5)	679	<10^−3^	234 (n = 5)	414	.15
p-JNK	0 (n = 10)	241	3897 (n = 5)	1561	.002	−292 (n = 5)	804	.77	0 (n = 10)	374	2701 (n = 5)	1119	.002	605 (n = 5)	280	.003
P65	0 (n = 8)	356	−505 (n = 5)	725	.90	−76 (n = 4)	700	.58	0 (n = 9)	359	−1051 (n = 5)	1151	.95	969 (n = 5)	423	.002

<I>, bias-corrected mean signal intensity (see Methods) for a given condition, with number of spots of that condition on the chip. SE, standard error of mean signal intensity for a given condition. Pval, p-value of difference between condition <I> and control <I>, measured by one-tailed, two-sample T-test.

To explore the utility of cell chip technology for pathway analysis, we examined the chip's ability to recapitulate the interferon response of A549 cells. Exposure to interferon activates the JAK/STAT signal transduction cascade, resulting in up-regulation of interferon response genes, including dsRNA-activated protein kinase (PKR), the 2′–5′ oligoadenylate synthetases (OAS), and the Myoxovirus resistance gene (Mx) [25]. We chose two assay targets, PKR and phospho-STAT1, to further validate the accuracy of the cell chip technology and to explore its dynamic range. We grew cells and exposed them to IFN-α (1000 U/ml) for 15 minutes or 18 hours before trypsinizing and formaldehyde fixing cells, along with untreated control cells. Technical and biological repeats were printed on the same slide (‘print 1’). At the same time, an equal number of cultures were prepared but stored in −20°C methanol for seven weeks before printing in an identical manner (‘print 2’). After printing, one slide from each print was immunoprobed for phosphorylated STAT1, counterstained with nuclear stain, and each spot was imaged at 40X. A second slide from each print was probed for PKR.

**Figure 3c** shows representative nuclear stain and immunofluorescence images of an individual spot from a slide probed for phospho-STAT1. Results of quantitative analysis are shown in **Table 2**. A fifteen-minute interferon exposure gave very strong signal for both prints, as well as weaker signal after 18 hours, indicating no loss of signal due to storage of fixed cells prior to printing. A third chip was probed after 30 days of storage at 4°; it showed less overall signal strength across all spots but also less variance, resulting in p-values nearly identical to the other two chips.

**Table 2:** Measuring cellular response to interferon treatment and signal variance due to experimental methods.

Probe	Slide	Control		Interferon α (1000 U/ml)					
				15 min			18 hr		
		<I>	SE	<I>	SE	Pval	<I>	SE	Pval
p-STAT1	Print 1	0 (n = 20)	47	860 (n = 10)	278	<10^−5^	115 (n = 9)	143	.02
	Print 2	0 (n = 19)	102	3100 (n = 10)	1056	<10^−5^	153 (n = 10)	71	<10^−4^
	Print 2 - Stored	0 (n = 19)	64	423 (n = 10)	118	<10^−6^	88 (n = 10)	57	<10^−3^
PKR	Print 1	0 (n = 17)	246	83 (n = 9)	232	.20	620 (n = 8)	414	.002
	Print 2	0 (n = 20)	278	156 (n = 10)	148	.03	416 (n = 10)	228	<10^−3^

P-values of each condition are shown. Print 1: normal cell prep, immediate printing and assay. Print 2: Cells fixed and stored for seven weeks at −20°C before printing. Stored: Cell chip stored for 30 days at 4°C after printing, before probing. For column headers, see legend for Table 1.

The slides probed for PKR showed a small increase in signal at the 18-hour timepoint (**Figure 2d**; **Table 2**), but, as expected, no response in the 15-minute samples. The small increase probably reflects the fact that PKR under these conditions is only up-regulated ∼3-fold (measured by Western blotting; data not shown). This low relative signal may bound the sensitivity of the current state of this technology.

The presence of multiple cell lines and treatment conditions on the same slide can be exploited as internal controls for both experimental conditions and probes. In the anisomycin/TNFα chips, for example, p38 kinase showed lower response to anisomycin treatment in A549 cells than in HeLa. The anti-phospho-p38 antibody gave the expected response for the HeLa cells on the same slide, which serves as a positive control for the probe. JNK kinase responded to anisomycin in both the A549 and HeLa cells, which are drawn from the same population as those probed for phospho-p38, indicating the drug treatment worked properly. Therefore it is reasonable to conclude that the observed difference in p38 activation reflects a biological phenomenon rather than an experimental artifact. This property of multiplex controls can in principle be applied to much larger screens including a wider variety of experimental conditions and probes.

## Discussion

We have demonstrated the capability of the cell chip to probe multiple aspects of cellular state using a variety of cell types and treatment conditions. Since cells are grown and treated under standard tissue culture conditions, treatment protocols such as transfections or drug exposure times can be optimized for each set of samples individually without affecting the assay. Furthermore, the adaptor molecule used to bind cells to the slide, WGA-biotin, targets a broad spectrum of human and other cell lines, but even this step could be optimized on a per-cell-line basis by using a specific biotinylated lectin. Also, the ability to store cells prior to printing allows researchers to perform large library transfections or other treatments asynchronously rather than all at once immediately before printing. Finally, although we analyzed images to gather population data across the cells in a printed spot, it is clear that single-cell data could be gleaned using more sophisticated image processing techniques [26], [27]. For transfection experiments, adding optical reporters (e.g., green fluorescent protein) on the same expression vector as the clone of interest or by co-transfection allows the measurement of cellular response exclusively on successfully transfected cells [16], mitigating the signal loss encountered when low transfection efficiency is averaged over a population.

The cell chip is complementary to, and in some cases an advance over, current high-throughput cell-based assay technologies. It differs from transfected cell arrays [11] in that it allows the analysis of multiple cell types and multiple growth and treatment conditions on a single slide, and it offers an order of magnitude increase in sample density over existing tissue microarray technology [17]. Finally, in probing samples from diverse populations for a single reporter, the cell chip represents an orthogonal assay to the single-population, many-reporter gene expression DNA microarray.

A key constraint of the technology as described here is the considerable manual effort required to prepare a source plate for printing. While we consider it a major advantage of the cell chip that cells are grown under normal tissue culture conditions, pin-based printing requires very high cell density in the source plate, which is not easily achieved by automated cell handling techniques. The use of other printing technologies, for example inkjet or other microspray methods, might ease this burden and make microplate-based cell culture growth and treatments more compatible with cell chip printing.

Such development of cell chip technology could make it readily applicable to functional genomics and chemogenomics. With the ability to probe an array of cells for target protein abundance, activation state, and subcellular localization, libraries of small molecule effectors could be screened for their impact on a variety of cellular systems. Furthermore, the technology could in principle be adapted for on-chip fluorescence *in-situ* hybridization (FISH) assays against nucleic acid targets. The fact that multiple dosages and timepoints can be printed on the same set of cell chips increases the depth to which researchers can investigate the impact of chemical libraries. Likewise, whereas typical functional genetic screens are designed around a single reporter or phenotype, the cell chip allows a different reporter for each replicate slide. Thus, each genetic perturbation could be assayed for impact on multiple cellular subsystems and/or for multiple reporters within the same system, greatly multiplying the data “bang” for the experimental “buck.” Taking into account all these features, we believe the cell chip offers a useful and general approach for medium- to large-scale cell-based assays.

## Supporting Information

Table S1 Supporting Data. Per-spot bias-corrected mean signal intensity data for each probed chip.
(0.02 MB XLS)
